# Cloning and characterization of a mannose binding C-type lectin gene from salivary gland of *Aedes albopictus*

**DOI:** 10.1186/1756-3305-7-337

**Published:** 2014-07-22

**Authors:** Jinzhi Cheng, Yu Wang, Fangzhan Li, Jian Liu, Yu Sun, Jiahong Wu

**Affiliations:** 1 Department of Parasitology, Guiyang Medical College, Guiyang, Guizhou 550004, China; 2 Lab for Modern Pathogen Biology, Guiyang Medical College, Guiyang, Guizhou 550004, China; 3 Guizhou Center for Disease Control and Prevention, Guiyang, Guizhou 550004, China; 4 Affiliated Hospital of Guiyang Medical College, Guiyang, Guizhou 550004, China

**Keywords:** C-type lectin, Salivary gland, *Ae. albopictus*, Prokaryotic expression, Agglutinating activity

## Abstract

**Background:**

The studies on sialomes have shown that hematophagous mosquito saliva consists of a lot of pharmacologically active proteins, in which C-type lectins have been identified and regarded as an important component of saliva. The previous studies showed that C-type lectins play crucial roles not only in innate immunity but also in promoting disease transmission in mammals. However, the function and mechanism of C-type lectins from the mosquito sialome is still elusive.

**Methods:**

A putative C-type lectin gene (*Aalb_CTL1*) was cloned and expressed from *Aedes albopictus* by RT-PCR. The deduced amino acid sequence was analyzed by bioinformatic methods. The gene expression profiles in different tissues and various blood-fed stages of *Ae. albopictus* were examined by Real-Time qRT-PCR and the biological functions of the recombined mature Aalb_CTL1 were tested by hemagglutination and sugar inhibitory agglutination assays. Moreover, the capabilities of rAalb_CTL1 against microorganisms were measured by microbial-agglutination assay.

**Results:**

The full-length Open reading frame (ORF) of *Aalb_CTL1* consisted of 462 bp, encoding 153 amino acid residues. The deduced amino acid sequence contained a putative signal peptide of 19 amino acids. It also contained a CRD domain with a WND (Trp^137^-Asn^138^-Asp-^139^) motif that needed calcium for the hemagglutinating activity and an imperfect EPS (Glu^128^-Pro^129^-Ser^130^) motif that had a predicted ligand binding specificity for mannose. The mRNA level of *Aalb_CTL1* was much higher in female mosquito salivary gland than those in fat body and midgut which was down-regulated in salivary gland after blood feeding. The rAalb_CTL1 contained not only hemagglutinating activity and a high affinity with mannose but also agglutinating activity against yeast *C. albicans* and Gram-positive bacteria *S. aureus* in Ca^2+^ dependent manner.

**Conclusion:**

Aalb_CTL1 was a mannose-binding C-type lectin and constituted one of the important components in saliva of *Ae. albopictus,* which could be involved in the defense against yeast and Gram-positive bacteria infection*.*

## Background

C-type lectins (CTLs) constitute the largest and most diverse super family of proteins that share a characteristic carbohydrate recognition domain (CRD) with two or three pairs of disulfide bonds [1,2]. Due to their ability to bind to specific carbohydrates on the surface of microorganisms, CTLs have been regarded as primary candidates for pattern recognition receptors (PRRs) to mediate pathogen recognition, which play an important role in the clearance of pathogens in innate immunity in vertebrates [3,4]. In invertebrates, the previous studies demonstrated that CTLs are also involved in innate immune responses, including the promotion of phagocytosis [5], nodule formation, encapsulation, melanization [6,7] and the activation of prophenoloxidase [8].

Mosquitoes are one of the most important medical arthropods. The harm to human beings caused by mosquitoes are not only in its harassment and blood-feeding habits, but also in its transmission of various diseases, such as malaria, dengue fever, Japanese encephalitis, yellow fever, Venezuelan equine encephalitis and Ross River fever [9]. The whole genomics studies demonstrated that there are 25, 39 and 55 CTLs respectively in *Anopheles gambiae*[10], *Aedes aegypti*[11] and *Culex quinquefasciatus*[12], which mostly are soluble proteins containing a single CRD without accessory domains. Several CTLs have exhibited a diverse range of function in mosquito innate immunity. The CTL4 and CTLMA2 from malaria vector *An. gambiae* can inhibit *Plasmodium berghei* ookinete melanization, indicating that they act as agonists of *Plasmodium* development in the vector [13]. In *Ae. aegypti*, a C-type lectin ( AaegmosGCTL-1 ) induced by West Nile Virus (WNV) infection, can interact with WNV in a calcium-dependent manner, and facilitate flavivirus invasion *in vivo* and *in vitro*[14]. By *in vivo* reverse genetic analysis, 9 mosGCTL genes from *Ae. aegypti* are identified as important susceptible factors to produce DENV-2 infection, of which mosGCTL-3 exhibits the most significant effect [15]. Based on the evidence above, it can be suggested that mosquito CTLs could be used as ligands by pathogens to promote the infection of a vector. On the other hand, the CTL4 and CTLMA2 have also been found to be required for the clearance of *Escherichia coli*, but not *Staphylococcus aureus*, suggesting that they play a role in fighting against Gram-negative bacteria infection [16]. Therefore, it could be inferred that the CTLs from mosquitoes could serve pleiotropic functions in the interaction of mosquito and pathogen.

*Ae. albopictus* is an efficient laboratory vector for a large number of arboviruses such as dengue virus, yellow fever virus, West Nile virus and several others. In China, it is also an important vector to transmit dengue fever. In *Ae. albopictus*, two CTLs, AY826070 and AY806069, have been identified by sialotranscriptome analysis [17]. In this study, the open reading frame (ORF) of AY826070 from *Ae. albopictus* Guangzhou strain (named *Aalb_CTL1*) was cloned by RT-PCR and expressed with prokaryotic expression system. The results of Real-Time quantitative RT-PCR (Real-Time qRT-PCR) showed that *Aalb_CTL1* was expressed specifically in female mosquito salivary gland. rAalb_CTL1 demonstrated agglutinating activity against animal erythrocyte, Gram-positive bacteria and yeast. It laid a preliminary foundation to make further study on the role of Aalb_CTL1 in salivary immunity.

## Methods

### Mosquitoes and sample collection

*Ae. albopictus* mosquitoes (Guangzhou strain) were kindly provided by Prof. Zhao Tongyan from Beijing Institute of Microbiology and Epidemiology and routinely reared at 25 ± 1°C with 75-80% relative humidity and a 14:10 (light: dark) photoperiod. Mosquitoes were fed on anesthetized mice.

Tissue samples including salivary glands, midgut and fat body from the sugar-fed adult female mosquitoes at 3–5 days old were dissected in the normal saline (NS: 0.9% NaCl, pH 6.5) and immediately soaked in the TRIzol Reagent (Invitrogen Life Technologies) and stored at −80°C until use. Six biological replicates, each consisting of salivary glands from 6 mosquitoes, midgut and fat body from 1 mosquito, were collected for each sample.

Salivary glands were further dissected at various time points: 3–5 days post emergence (PE) and 0 h, 24 h and 72 h post blood-feeding. Mosquitoes, 3–5 days PE, were fed on anesthetized mice for 2 h, and the mosquitoes with engorged abdomen were selected and regarded as 0 h post blood-feeding. Six biological replicates, each consisting of salivary glands from 6 mosquitoes, were collected for each time point.

### Full-length ORF cloning of *Aalb_CTL1*

Total RNA was extracted from female mosquito with TRIzol Reagent according to the manufacturer’s protocol. The single-stranded cDNA was synthesized using PrimeScript™ RT Kit (TaKaRa). *Aalb_CTL1* specific primers for full length ORF (Table 1) were designed according to the published cDNA sequence of *Ae. albopictus* [GenBank: AY826070]. The desired products were amplified by 35 cycles of PCR using 2 μl synthesized cDNA as template in 50 μl reaction containing 5 μl 10 × Ex Taq buffer (containing Mg^2+^), 40pmol each primer and 5 U Ex Taq DNA Polymerase (TaKaRa). The PCR conditions were 5 min at 94°C for one cycle, followed by 35 cycles of denaturation at 94°C for 30 sec, annealing at 58°C for 30 sec, and extension at 72°C for 1 min. In the last cycle, the PCR product was further incubated at 72°C for 7 min to allow the completion of DNA synthesis.

**Table 1:** Primers used in present study

**Gene name**	**Forward primer (5′-3′)**	**Reverse primer (5′-3′)**	**Amplicon size (bp)**
*Aalb_CTL1* ORF cloning			
*Aalb_CTL1*	ATGGGGCAGCCATCATCATCATCAT	ACATTCGTTCGCACACAAAAC	462
Recombinant expression			
*Aalb_CTL1*	GTC*CATATG*CAACAGGAATGCGACTGTAAAAATAG	GTG*AAGCTT*ACATTCGTTCGCACACAAAAC	405
Quantitative real-time PCR (Q-PCR)			
*Aalb_CTL1*	GCTATCTCTCTCGCAGACCTCAC	CCAATCTCCAGCCGTTCCT	130
Rsp 5	ATTACATCGCCGTCAAGGAG	TCATCATCAGCGAGTTGGTC	126

The restriction endonuclease sites in italics.

The amplified products were purified using DNA purification kit (TaKaRa). The purified PCR products were cloned into the pMD18-T cloning vector (TaKaRa) at mole ratio 5:1 at 16°C and then transformed into freshly prepared *E. coli* strain DH5α competent cells. Recombinant clones were selected using blue/white screening on X-gal (25 μg/mg)/IPTG (25 μg/ml)/Ampicillin (50 μg/ml) LB plates. The clones were confirmed by *Nde I and Hind III* restriction enzymes digestion. Three positive clones were picked up for sequencing in both directions.

### Sequences analysis

The homology analyses of the nucleotides and deduced amino acid sequences of *Aalb_CTL1* were carried out using the BLAST programs at the NCBI (http://www.ncbi.nlm.nih.gov/blast). Translation of the cDNA was performed using the Expert Protein Analysis System (http://au.expasy.org). Signal peptide and CRD domain predictions were conducted using SMART software (http://smart.embl-heidelberg.de/). The homologous sequences from various species were downloaded from NCBI. A phylogenetic tree was constructed using the Neighbour-joining method with the Molecular Evolutionary Genetics Analysis (MEGA 4) package.

Real-Time quantitative Reverse Transcription Polymerase Chain Reaction **(Real-Time qRT-PCR)** analysis.

Total RNA was extracted for all the tissue samples separately with TRIzol Reagent according to the manufacturer’s protocol. Before reverse transcription, the RNA samples were treated with DNAase I (Invitrogen) and the single-stranded cDNAs were synthesized using PrimeScript™ RT Kit (TaKaRa). According to the cDNA full-length sequence, one pair of gene-specific primers for *Aalb_CTL1* (Table 1) was used to amplify product of 130 bp from the cDNA. Two rsp5 primers (Table 1) were used to amplify a 126 bp fragment as an internal control to verify the successful transcription and to calibrate the cDNA template for corresponding mosquito samples [18].

Real-Time PCR amplification was carried out in an ABI 7300 Real-Time Thermal Cycler according to the manual (TaKaRa). Dissociation curve analysis of amplification products was performed at the end of each PCR to confirm that only one PCR product was amplified and detected. The two/double standard curve method was used to analyze the expression level of *Aalb_CTL1*. All data were given in terms of mRNA expressed as means ± SE. Significant differences between means were determined using one way ANOVA and P < 0.05 was considered statistically significant.

### Prokaryotic expression and purification of recombinant Aalb_CTL1

A cDNA fragment encoding a mature Aalb_CTL1 protein (residues 20 to 153) was amplified by PCR using Ex Taq polymerase (TaKaRa) with the specific primers for recombinant expression (Table 1). *Nde I and Hind III* restriction sites were added to the 5′ ends of *Aalb_CTL1* primers (after the stop codon), respectively. The PCR fragment was cloned into the pMD18-T vector (TaRaKa) firstly, it was completely digested with *Nde I and Hind III* (TaRaKa), and cloned into the *Nde I and Hind III* sites of the expression vector pET28a(+) subsequently, then the recombinant plasmid (pET28-*Aalb_CTL1*) was transformed into *E. coli* BL21 (DE3). Positive clones were screened by enzymatic digestion with *Nde I and Hind III* and confirmed by nucleotide sequencing. Finally, positive transformants were incubated in 100 ml LB medium (10 g tryptone,5 g yeast extract, 10 g NaCl/1 l distilled water, pH 7.0) at 37°C with shaking at 220 rpm until the culture reached OD600 of 0.5-0.7, and 1 mmol/L isopropyl-β-D-thiogalactosidase (IPTG) was added to the medium under the same conditions for another 5 h. Bacterial cells expressing recombinant proteins were harvested and sonicated, and the inclusion bodies were resuspended in 1 × phosphate-buffered saline (PBS: NaCl 137 mmol/L, KCl 2.7 mmol/L, Na_2_HPO_4_ 10 mmol/L, KH_2_PO_4_ 2 mmol/L, pH 7.4) containing 8 M urea. Recombinant Aalb_CTL1 protein (rAalb_CTL1) was purified by gradient dialysis at 4°C overnight, and further purified with His•Bind Purification Kit (Novagen) according to the manufacturer’s instructions. Purified recombinant protein was dialyzed against 1 × PBS buffer at 4°C overnight. The resulting protein was analyzed by 15% sodium dodecyl sulfate-polyacrylamide gel electrophoresis (SDS-PAGE) and visualized with Coomassie brilliant blue R-250.

### Hemagglutination assays

To test whether or not calcium was required for the hemagglutinating activity of rAalb_CTL1, 12.5 μl of serial dilutions of CaCl_2_ in NS was mixed with 12.5 μl of rAalb_CTL1 (~50 μg/ml). BSA instead of Aalb_CTL1 was used as a negative control. After 25 μl 2% suspension of mouse erythrocytes was added, the mixture was incubated for 30 min at 25°C. Hemagglutination was observed under a microscope (Nikon TE2000, Japan). Assays were performed in triplicate independently.

The hemagglutinating activity of rAalb_CTL1 was tested using 2% (vol/vol) erythrocytes from rabbits, mice, and rats according to the method described by Luo *et al.*[19]. Erythrocytes were washed five times with NS-Ca buffer (0.9% NaCl,40 mM CaCl_2_, pH 6.5) and then diluted with NS-Ca buffer to 2% (vol/vol) suspension. Two-fold serial dilutions (25 μl) of rAalb_CTL1 in NS-Ca buffer were mixed with 25 μl of the erythrocyte suspension in a microtiter V plate. The plate was incubated for 1 h at room temperature, and the hemagglutination was observed under a microscope (Nikon TE2000, Japan). Erythrocyte suspension mixed with serial dilutions of bovine serum albumin (BSA, Sigma) in NS-Ca buffer were processed in parallel to serve as negative controls.

### Sugar binding specificity of rAalb_CTL1

The sugar binding specificity of rAalb_CTL1 was determined by an inhibitory agglutination assay. Serial dilutions (12.5 μl) of various carbohydrates (Sigma) in NS-Ca buffer, including D-mannose, D-galactose, Lactose, D-glucose, Maltose and Sucrose, were mixed with 12.5 μl of rAalb_CTL1 (12 μg/ml) and incubated for 30 min at 37°C. Then, 2% erythrocytes suspension was added, and the mixture was incubated for 30 min at room temperature. An inhibitory effect was observed at the minimum concentration of a carbohydrate required for complete inhibition of the hemagglutinating activity of rAalb_CTL1. This assay was performed in triplicate independently.

### Microbial-agglutination assays

Three microorganisms were used for agglutination tests including yeast *C. albicans* (ATCC10231), Gram-negative bacteria *E. coli* (ATCC25922) and Gram-positive bacteria *S. aureus* (ATCC25923). All three microorganisms were obtained from Beijing Institute of Microbiology and Epidemiology and maintained in our laboratory. Bacteria were streaked on LB agar plates at 37°C for 18 h firstly, then a single colony was inoculated into 5 ml LB medium and cultured overnight at 37°C. The bacteria were cultured again in fresh medium at 1:100 dilution for 5 h at 37°C and collected in mid logarithmic phase by centrifugation at 6000 rpm for 5 min.

*C. albicans* were streaked in Sabouraud’s agar plate (10 g tryptone, 40 g glucose/1 l distilled water, pH 5.6; with 20 g agar) at 25°C for 24 h, then a single colony was inoculated into 5 ml Sabouraud’s medium and cultured for 36 h at 25°C. The yeast was cultured again in fresh Sabouraud’s medium at 1:100 dilution for 24 h at 25°C and collected by centrifugation at 6000 rpm for 5 min.

The yeast or the bacteria prepared as above were stained by DAPI for 20 min, washed twice with TBS buffer (Tris 50 mmol/L, NaCl 150 mmol/L, pH 7.6) and resuspended in TBS buffer at a concentration of 2 × 10^8^ cells/ml. Then 50 μl TBS including rAalb_CTL1 at final concentration 50 μg/ml was added into 50 μl of bacteria/yeast. After incubation for 1 h at room temperature in the presence or absence of 40 mM CaCl_2_, these samples were observed and photographed under a fluorescence microscope (Nikon TE2000, Japan). BSA instead of rAalb_CTL1 was used as a negative control (BSA-Ca control).

### Ethical approval

Ethical approval documents were given by the Experimental Animal Ethics Committee of Guiyang Medical College.

## Results and discussion

Saliva of mosquitoes consists of a milieu of pharmacologically active substances to function not only in food uptake but also in disease transmission [20]. Sialome studies have shown that there are at least 100 proteins in mosquito saliva, and more than one CTL has been identified and predicted in mosquito sialomes [17,21,22]. However, little is known about the functions of them. In our study, we detected the agglutinating activity of Aalb_CTL1.

### cDNA cloning and sequence analysis of *Aalb_CTL1*

**Figure 1:**
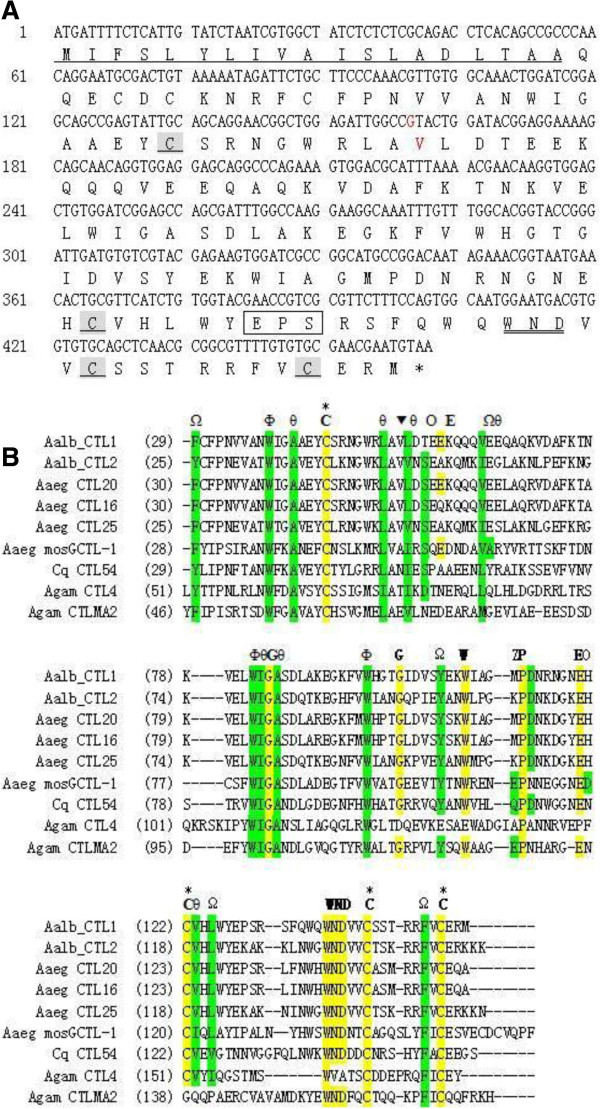
**Sequence analysis of Aalb_CTL1. (A)** ORF and deduced amino acid sequence of *Aalb_CTL1* from the *Aedes albopictus* Guangzhou strain. The amino acid sequence is represented by a single capital letter below the nucleotide sequence. The putative signal peptide sequence is underlined; four conserved cysteine residues that define the C-type lectin domain are shaded and underlined. The EPS motif for ligand binding specificity is in box. The WND motif is in double lines. The mutation position is in red. **(B)** Comparison of a putative CRD of Aalb_CTL1 with known and putative mosquito C-type lectins. Alignment of the CRD domain from Aalb_CTL1, Aaeg CTL16 [VectorBase: AAEL000533-RA], Aaeg CTL20 [VectorBase: AAEL011407-RA], Aaeg CTL25 [VectorBase: AAEL000556-RA], Aaeg mosGCTL-1 [VectorBase: AAEL000563-RA], Aalb_CTL2 [Genbank: AAV90641], Cq CTL54 [VectorBase: CPIJ014105], Agam CTL4 [VectorBase: ENSANGG00000018677], Agam CTLMA2 [VectorBase: ENSANGG00000018421]. Invariant or highly conserved residues within the CRD [Drickamer, 1993] are shown in the upper row: the italic single-letter amino acid codes indicate invariant conserved amino acids. Other abbreviations: Θ, aliphatic; Φ, aromatic, Ω aliphatic or aromatic; O, oxygen-containing; Z, Glutamine or glutamic acid; triangle: the position for mutation. Cysteine residues forming disulphide bonds are marked with asterisks. Numbers on the left indicate amino acid positions starting from the initial methionine.

Based on the cDNA sequence of *Aalb_CTL1*, gene-specific primers were designed and the ORF of *Aalb_CTL1* was cloned and sequenced from the *Ae. albopictus* Guangzhou strain. A total of 462 bp encoding a protein of 153 amino acids was obtained (Figure 1A). Sequence alignment showed there was a base mutation at position 160 from A to G, which in turn caused an amino acid change from I to V in the *Ae. albopictus* Guangzhou strain. Multiple sequence alignment of carbohydrate recognition domain (CRDs) from *Ae.aegypti* and *Cx. quinquefasciatus* demonstrated that V was more conservative than I in this position (Figure 1B). It is well known that both V and I belong to the aliphatic amino acids, but a further study would be performed to test the effect on function of Aalb_CTL1 with such an amino acid change.

According to the ORF of *Aalb_CTL1*, the deduced amino acids sequence contained a putative signal peptide of 19 residues at the N-terminal, suggesting that it should be a secreted protein and would be a component of saliva in mosquito. The calculated molecular mass of the mature Aalb_CTL1 protein (residues 19 to 153) is 15.71 kDa, with an estimated pI of 5.72. Like other C-type lectins, Aalb_CTL1 had a single characteristic CRD stabilized by two highly conserved disulphide bridges from four cysteine residues Cys 45, Cys 122, Cys 142 and Cys 150 [23] (Figure 1A). Multiple sequence alignment of CRDs (Figure 1B) demonstrated that the CRD of Aalb_CTL1 contained 13 of 14 invariant and 14 of 18 highly conserved amino acid residues, which were identified as a C-type CRD by Drickamer *et al*. [23] and Yamanoto-Kihara *et al.*[24]. A WND motif (Trp137-Asp138-Asn139) was identified in the C-terminal of CRD of Aalb_CTL1, indicating that calcium was important for Aalb_CTL1 to possess hemagglutinating activity [25]. In addition to WND motif, an imperfect EPS motif (Glu^128^-Pro^129^-Ser^130^) was also present in the CRD of Aalb_CTL1. For a C-type lectin of uncharacterized function, it can be predicted that CRDs containing a WND motif and an EPN motif might be involved in carbohydrate-binding, whereas, the specificity for either mannose or galactose depends on whether the motif is EPN or QPD [25]. Therefore, it could be further postulated that Aalb_CTL1 might be mannose-specific though the EPN was substituted by EPS in Aalb_CTL1.

**Figure 2:**
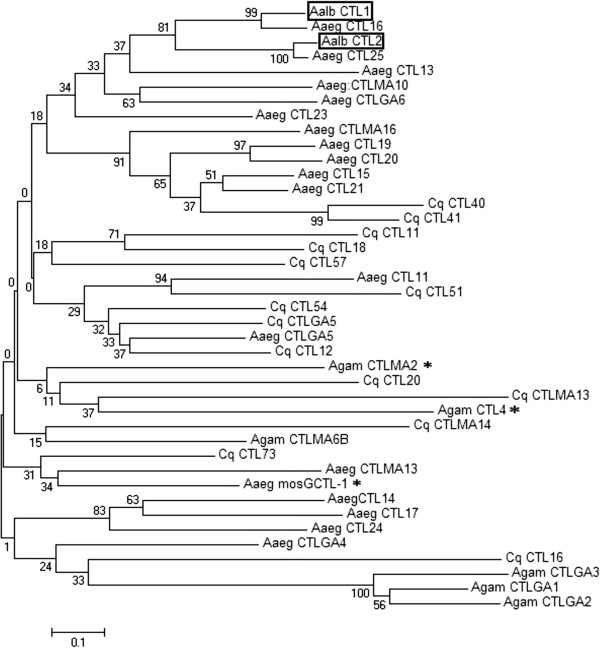
**Phylogenetic tree constructed based on full-length amino acids sequence of Aalb_CTL1 and putative or known C-type lectins from mosquitoes.** The C-type lectin sequences were from Aaeg CTL11 [VectorBase: AAEL008299], Aaeg CTL13 [VectorBase: AAEL004679], Aaeg CTLGA4 [VectorBase: N41092], Aaeg CTL14 [VectorBase: AAEL011453], Aaeg CTLGA5 [VectorBase: AAEL005641-RA], Aaeg CTLMA10 [VectorBase: AAEL011079-RA], Aaeg CTL15 [VectorBase: AAEL012353-RA], Aaeg CTLGA6 [VectorBase: AAEL009209], Aaeg CTL17 [VectorBase: AAEL011446-RA], Aaeg CTL19 [VectorBase: AAEL011404-RA], Aaeg CTLMA13 [VectorBase: AAEL011621-RA], Aaeg CTL20 [VectorBase: AAEL011407-RA], Aaeg CTL21 [VectorBase: AAEL011408-RA], Aaeg CTL23 [VectorBase: AAEL006456-RA], Aaeg CTL24 [VectorBase: AAEL002524-RA], Aaeg CTL25 [VectorBase: AAEL000556-RA], Aaeg mosGCTL-1 [VectorBase: AAEL000563-RA], Aaeg CTLMA16 [VectorBase: AAEL000283-RA], Aaeg CTL16 [VectorBase: AAEL000533-RA], Cq_CTL11[VectorBase: CPIJ000449], Cq_CTL12 [VectorBase: CPIJ001323], Cq_CTL16 [VectorBase: CPIJ003650], Cq_CTL18 [VectorBase: CPIJ004339], Cq_CTL20 [VectorBase: CPIJ004916], Cq_CTLMA13 [VectorBase: CPIJ007062], Cq_CTL40 [VectorBase: CPIJ007868], Cq_CTL41 [VectorBase: CPIJ007869], Cq_CTL51 [VectorBase: CPIJ012307], Cq_CTL54 [VectorBase: CPIJ014105], Cq_CTL57 [VectorBase: CPIJ015095], Cq_CTLMA14 [VectorBase: CPIJ015742], Cq_CTL73 [VectorBase: CPIJ016688], Cq_CTLGA5 [VectorBase: CPIJ017075], Agam_CTLMA6B [VectorBase: ENSANGG00000018449], Agam_CTLMA2 [VectorBase: ENSANGG00000018421], Agam_CTL4 [VectorBase: ENSANGG00000018677], Agam_CTLGA1 [VectorBase: ENSANGG00000009790], Agam_CTLGA2 [VectorBase: ENSANGG00000017954], Agam_CTLGA3 [VectorBase: ENSANGG00000009745]. In the Phylogenetic analysis, Aalb_CTL1 and Aalb_CTL2 are in the box from *Ae. albopictus.* Agam CTLMA2, Agam CTL4 and Aaeg mosGCTL-1 whose function has been reported and are marked with*, The bar (0.1) indicates genetic distance.

Blast analysis with the deduced amino acid sequence of *Aalb_CTL1* revealed that Aalb_CTL1 shared a high degree of similarity with several known or putative C-type lectin family members from mosquitoes, especially the CTL from *Ae. aegypti* (Aaeg CTL16, similarity 83%). These mosquito CTLs contain only one CRD (Figure 1B), which is different from most of the CTLs from the Lepidopteran containing dual CRDs [26]. In order to understand the evolutionary relationship of Aalb_CTL1 in mosquitoes, a phylogenetic tree was constructed with the CTLs sequences from *Ae. aegypti*, *An. gambiae* and *Cx. quinquefasciatus* that was highly similar with Aalb_CTL1. In the phylogenetic tree (Figure 2), the Aalb_CTL1 was clustered with Aaeg CTL16 firstly, then clustered with Aalb_CTL2 and Aaeg CTL25, and they further formed a big branch with some CTLs from Culex subfamily. CTLs from Anopheles subfamily were clustered together with the other CTLs from Culex subfamily. The relationships displayed in the phylogenetic tree showed that Aalb_CTL1 could belong to Culex-specific proteins. The above-mentioned evidences in our data has supported the Batholomay’s notion [27] that Aaeg CTL16 locates in the upper part of the phylogenetic tree and no CTLs from *An. gambiae* and *Drosophila melanogaster* are clustered together with it.

### Tissue-specific expression and stage-dependent expression of *Aalb_CTL1*

**Figure 3:**
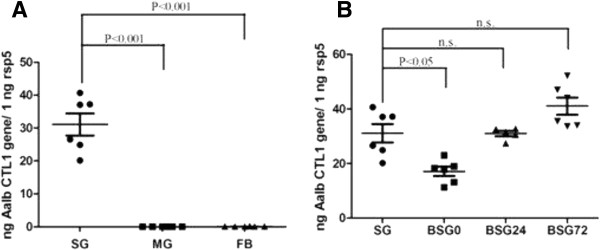
**Temporal and spatial expression profiles of *Aalb_CTL1 *(n = 6). (A)** tissue expression profile of *Aalb_CTL1* in *Ae. albopictus*. SG: salivary gland, MG: midgut, FB: fat body. **(B)** stage-dependent expression of *Aalb_CTL1* after blood-feeding. BSG_0: 0 time after engorgement; BSG_24: 24 hours after engorgement; BSG_72 hours after engorgement.

The tissue-specific expression and temporal expression pattern of *Aalb_CTL1* in the salivary gland after blood-feeding were determined by Real-Time qRT-PCR. The expression of *Aalb_CTL1* at transcription level was much higher in the salivary gland than those in the midgut and fat body (Figure 3A, P < 0.001), indicating that *Aalb_CTL1* would be expressed specifically in salivary gland and could be one of the important components in saliva as a secreted protein. *Aaeg CTL16* from *Ae. aegypti*, revealing a high degree of sequence identity with *Aalb_CTL1*, has been verified to be salivary gland-specific lectin by semi-quantitative RT-PCR [27]. The expression level of *Aalb_CTL1* was significantly down-regulated at 0 time after blood-feeding (Figure 3B, P < 0.05), suggesting that the expression of *Aalb_CTL1* transcript could be modulated by blood-feeding.

### Hemagglutinating activity (HA) and sugar binding specificity of rAalb_CTL1

**Figure 4:**
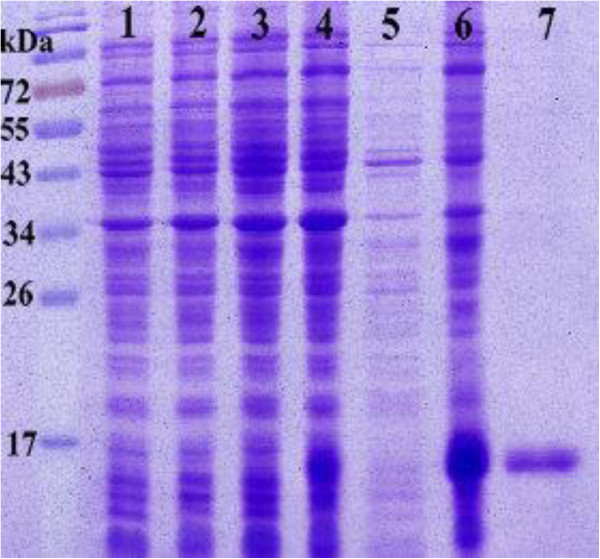
**Expression and purification of recombinant mature Aalb_CTL1 (rAalb_CTL1).** Lane M: protein molecular standard. Lane 1: pET-28a(+) in BL21, uninduced. Lane 2: pET-28a(+) in BL21, IPTG-induced for 5 h. Lane 3: pET-28a(+)-*Aalb_CTL1* in BL21, uninduced. Lane 4: pET-28a(+)-*Aalb_CTL1* in BL21, IPTG-induced for 5 h. Lane 5: Supernatants of lysate of BL21 cell containing pET-28a(+)-*Aalb_CTL1* induced with IPTG. Lane 6: Sediments of lysate of BL21 cell containing pET-28a(+)-*Aalb_CTL1* induced with IPTG. Lane 7: pET-28a(+)-*Aalb_CTL1* recombinant proteins purified by the affinity purification method.

It is well known that not all proteins containing C-type CRDs can actually bind carbohydrate or even calcium [25]. To test the biological function of Aalb_CTL1, a recombinant plasmid pET-28a-*Aalb_CTL1* was transformed and expressed in *E. coli* BL21 (DE3). One clear band of the rAalb_CTL1 with molecular mass of 17 kDa was detected by SDS-PAGE analysis (Figure 4). The hemagglutination assay showed that rAalb*_*CTL1 at 40 mM CaCl_2_ could induce the hemaggutination of rabbit erythrocytes, rat erythrocytes and mouse erythrocytes, and no agglutination was observed in the rAalb_CTL1 without CaCl_2_ group and BSA-Ca negative control under the same conditions. Furthermore, the agglutination was inhibited in the presence of EDTA, indicating that the agglutination of animal erythrocytes by rAalb*_*CTL1 was Ca^2+^-dependent. The agglutinating activity of rAalb_CTL1 for rabbit erythrocytes was higher than that for mouse and rat erythrocytes at a minimal agglutination concentration of 6.25 μg/ml, compared with the minimal agglutination concentration of 12.5 μg/ml for mouse erythrocytes and rat erythrocytes.

The carbohydrate-binding inhibitory hemagglutination assay demonstrated that the hemagglutinating activity of rAalb_CTL1 were inhibited by all the six types of sugars at different concentrations in a calcium-dependent way (Table 2), but among the carbohydrates tested, D-mannose inhibited the hemagglutinating activity of rAalb_CTL1 most effectively with a minimal concentration of 6.25 mM. The results suggested that rAalb_CTL1 might have a high ligand affinity to bind mannose, which was associated with their structural features.

**Table 2:** **Sugar binding specificities of rAalb *_ *** **CTL1**

**Sugar name**	**Minimal Inhibitory Concentration (MIC) (mM)**
D-Glucose	25
D-Mannose	6.25
D-Galactose	25
Lactose	50
Sucrose	50
Maltose	25

### Agglutination of rAalb*_*CTL1 to microbes

**Figure 5:**
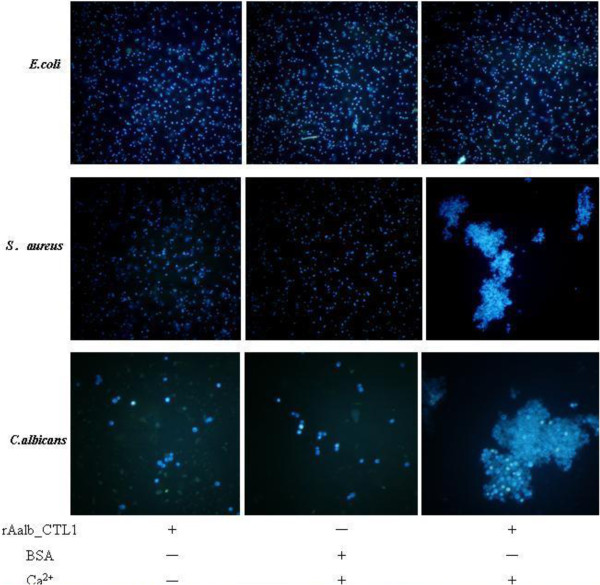
**Agglutination of three kinds of microorgnisms induced by rAalb_CTL1.** Gram-negative *E. coli,* Gram-positive *S. aureus* and yeast *C. albicans* shown (magnification 40×).

CTLs are pattern recognition receptors that can distinguish self from non-self in mammalians [28]. Many studies on insects demonstrated that CTLs were involved in the insect innate immunity either *in vivo* or *in vitro*, especially in Lepidopterans [29,30]. In the present study, to determine whether rAalb_CTL1 could induce the aggregation of microbial pathogens, DAPI-labeled yeast *C. albicans*, Gram-positive bacteria *S. aureus* and Gram-negative bacteria *E. coli* were incubated with the rAalb_CTL1. The rAalb_CTL1 displayed the activity to agglutinate yeast *C. albicans* and Gram-positive bacteria *S. aureus*, but no agglutinating activity toward Gram-negative bacteria *E. coli*. Meanwhile, there was also no agglutinating activity observed in the BSA-Ca control and the rAalb_CTL1 without calcium group, exhibiting that the agglutinating activity against yeast *C. albicans* and Gram-positive bacterium *S. aureus* was in a calcium-dependent fashion (Figure 5). The agglutinating activity of rAalb_CTL1 suggested that Aalb_CTL1 might be involved in eliminating pathogens from food. In the mosquito *Ae. aegypti*, immunity-related proteins expressed in salivary gland, such as lysozymes and C-type lectins, are postulated to have a role in controlling bacterial growth in sugar solutions stored in the crop or in the gut following a blood meal [21,31,32]. In addition, a previous study on CTLs of *An. gambiae* showed that CTL4 and CTLMA2 can recognize the surface molecules of Gram-negative *E. coli* but not Gram-positive *S. aureus*, which is different from that of rAalb_CTL1. Furthermore, the defensive function against Gram-negative bacteria is attributed to the CTL4-CTLMA2 heterodimer *in vivo*[16]. Thus, combined with the reasonable deduction of the result of the phylogenetic tree, it could be inferred that they might belong to the different members of CTLs and be involved in the recognition of different types of microorganisms.

## Conclusions

In this study, a putative C-type lectin (*Aalb-CTL1*) was successfully cloned and expressed from *Ae. albopictus* Guangzhou strain. Aalb_CTL1 was only clustered with CTLs from Culex subfamily in the phylogenetic tree, suggesting that it would be a Culex-specific protein. *Aalb_CTL1* was expressed specifically in salivary gland and its transcript level could be regulated by blood-feeding. rAalb-CTL1 exhibited not only the hemagglutinating activity and a high affinity for mannose but also the agglutinating activity against yeast *C. albicans* and Gram-positive bacteria *S. aureus* in a calcium-dependent manner. Taken together, our results showed that Aalb_CTL1 was a mannose-binding C-type lectin and could act as a PPR involved in the anti-microbial activity. More efforts should be focused on its role on the relationship of mosquito and pathogen in saliva in the future.

## Abbreviations

*Ae. albopictus*: *Aedes albopictus*; *Ae. aegypti*: *Aedes aegypti*; *An. gambiae*: *Anopheles gambiae*; *Cx. quinquefasciatus*: *Culex quinquefasciatus*; *E. coli*: *Escherichia coli*; *S. aureus*: *Staphylococcus aureus*; *C. albicans*: *Candida albicans*; CTLs: C-type lectins; Aalb_CTL1: C-type lectin 1 in salivary gland of *Ae. albopictus*; CRD: Carbohydrate recognition domain; PRRs: Pattern recognition receptors; IPTG: Isopropyl β-D-Thiogalactoside; SDS-PAGE: Sodium dodecyl sulfate polyacrylamide gel electrophoresis; BSA: Bovine serum albumin; Real-Time qRT-PCR: Real-Time quantitative reverse transcription polymerase chain reaction.

## Competing interests

All the authors declare that they have no competing interests.

## Authors’ contributions

CJZ participated in the experiment design, carried out the experiment and drafted the manuscript. WY analyzed the data and drafted the manuscript. LFZ and LJ carried out bioinformatics analysis and prokaryotic expression. SY collected the tissue samples including salivary gland, midgut and fat body. WJH conceived of the study, analyzed the data and wrote the manuscript. All authors read and approved the final version of the manuscript.

## References

[B1] Janeway CA Medzhitov R Innate immune recognition Annu Rev Immunol 2002 20 197 216 11861602 10.1146/annurev.immunol.20.083001.084359

[B2] Sharon N Lis H Lectins: cell-agglutinating and sugar-specific proteins Science 1972 177 949 959 5055944 10.1126/science.177.4053.949

[B3] Weis WI Taylor ME Drickamer K The C-type lectin superfamily in the immune system Immunol Rev 1998 163 19 34 9700499 10.1111/j.1600-065x.1998.tb01185.x

[B4] Hoffmann JA Kafatos FC Janeway CA Ezekowitz RA Phylogenetic perspectives in innate immunity Science 1999 284 1313 1318 10334979 10.1126/science.284.5418.1313

[B5] Tan YY Liu Y Zhao XF Wang JX Characterization of a C-type lectin from the cotton bollworm, *Helicoverpa armigera* Dev Comp Immunol 2009 33 772 779 19185587 10.1016/j.dci.2009.01.002

[B6] Koizumi N Imamura M Kadotani T Yaoi K Iwahana H Sato R The lipopolysaccharide-binding protein participating in haemocyte nodule formation in the silkworm *Bombyx moriis* is a novel member of the C-type lectin superfamily with two different tandem carbohydrate-recognition domains FEBS Lett 1999 443 139 143 9989592 10.1016/s0014-5793(98)01701-3

[B7] Yu XQ Kanost MR Immulectin-2, a pattern recognition receptor that stimulates hemocyte encapsulation and melanization in the tobacco hornworm, *Manduca sexta* Dev Comp Immunol 2004 28 891 900 15183030 10.1016/j.dci.2004.02.005

[B8] Yu XQ Kanost MR Immulectin-2, a lipopolysaccaride-specific lectin from an insect, Manduca sexta, is induced in response to gram-negative bacteria J Biol Chem 2000 275 37373 37381 10954704 10.1074/jbc.M003021200

[B9] Tan WB Wang X Cheng P Liu LJ Wang HF Gong MQ Quan X Gao HG Zhu CL Molecular cloning and preliminary function study of iron responsive element binding protein 1 gene from cypermethrin-resistant *Culex pipens pallens* Parasit Vectors 2011 4 215 doi:10.1186/1756-3305-4-215 22075242 10.1186/1756-3305-4-215 PMC3223502

[B10] Holt RA Subramanian GM Halpern A Sutton GG Charlab R Nusskern DR Wincker P Clark AG Ribeiro JM Wides R Salzberg SL Loftus B Yandell M Majoros WH Rusch DB Lai Z Kraft CL Abril JF Anthouard V Arensburger P Atkinson PW Baden H de Berardinis V Baldwin D Benes V Biedler J Blass C Bolanos R Boscus D Barnstead M The genome sequence of the malaria mosquito Anopheles gambiae Science 2002 298 129 149 12364791 10.1126/science.1076181

[B11] Nene V Wortman JR Lawson D Haas B Kodira C Tu ZJ Loftus B Xi Z Megy K Grabherr M Ren Q Zdobnov EM Lobo NF Campbell KS Brown SE Bonaldo MF Zhu J Sinkins SP Hogenkamp DG Amedeo P Arensburger P Atkinson PW Bidwell S Biedler J Birney E Bruggner RV Costas J Coy MR Crabtree J Crawford M Genome Sequence of Aedes aegypti, a major arbovirus vector Science 2007 316 1718 1723 17510324 10.1126/science.1138878 PMC2868357

[B12] Arensburger P Megy K Waterhouse RM Abrudan J Amedeo P Antelo B Bartholomay L Bidwell S Caler E Camara F Campbell CL Campbell KS Casola C Castro MT Chandramouliswaran I Chapman SB Christley S Costas J Eisenstadt E Feschotte C Fraser-Liggett C Guigo R Haas B Hammond M Hansson BS Hemingway J Hill SR Howarth C Ignell R Kennedy RC Sequencing of Culex quinquefasciatus establishes a platform for mosquito comparative genomics Science 2010 30 86 88 20929810 10.1126/science.1191864 PMC3740384

[B13] Mike AO George KC Fotis CK Effects of mosquito genes on plasmodium development Science 2004 303 2030 2032 15044804 10.1126/science.1091789

[B14] Cheng G Cox J Wang P Krishnan MN Dai J Qian F Anderson JF Fikrig E A C-type lectin collaborates with a CD45 phosphatase homolog to facilitate West Nile virus infection of mosquitoes Cell 2010 142 714 725 doi:10.1016/j.cell.2010.07.038 20797779 10.1016/j.cell.2010.07.038 PMC2954371

[B15] Liu Y Zhang FC Liu JY Xiao XP Zhang SY Qin CF Xiang Y Wang PH Cheng G Transmission-blocking antibodies against mosquito C-type lectins for Dengue prevention Plos Pathog 2014 10 e1003931 24550728 10.1371/journal.ppat.1003931 PMC3923773

[B16] Schnitger AK Yassine H Kafator FC Osta MA Two C-type lectins cooperate to defend *Anopheles gambiae* against Gram-negative bacteria J Biol Chem 2009 284 17616 17624 19380589 10.1074/jbc.M808298200 PMC2719400

[B17] Arcà B Lombardo F Francischetti IM Pham VM Mestres-Simon M Andersen JF Ribeiro JM An insight into the sialome of the adult female mosquito *Aedes albopictus* Insect Biochem Mol Biol 2007 37 107 127 17244540 10.1016/j.ibmb.2006.10.007

[B18] Wu JH Cheng JZ Sun Y Chen L Selection of control genes in Real-time qPCR analysis of gene expression in *Aedes albopictus* Chinese J Zoonoses 2011 17 432 435 (in Chinese )

[B19] Luo T Yang H Li F Zhang X Xu X Purification, characterization and cDNA cloning of a novel lipopolysaccharide-binding lectin from the shrimp *Penaeus monodon* Dev Comp Immunol 2006 30 607 617 16364436 10.1016/j.dci.2005.10.004

[B20] Champagne DE Antihemostatic strategies of blood-feeding arthropods Curr Drug Targets 2004 4 375 396 10.2174/1568006043335862 15578959

[B21] Ribeiro JM Arca B Lombardo F Calvo E Phan VM Chandra PK Wikel SK An annotated catalogue of salivary gland transcripts in the adult female mosquito, *Aedes aegypti* BMC Genomics 2007 8 1 6 doi:10.1186/1471-2164-8-6 17204158 10.1186/1471-2164-8-6 PMC1790711

[B22] Arca B Lombardo F Valenzuela JG Francischetti IM Marinotti O Coluzzi M Ribeiro JM An updated catalogue of salivary gland transcripts in the adult female mosquito, *Anopheles gambiae* J Exp Biol 2005 208 Pt 20 3971 3986 16215223 10.1242/jeb.01849

[B23] Drickamer K Ca2+−dependent carbohydrate-recognition domains in animal proteins Curr Opin Struct Biol 1993 3 393 400

[B24] Yamamoto-Kihara M Kotani E Isolation and characterization of a C-type lectin cDNA specifically expressed in the tip of mouthparts of the flesh fly *Sarcophaga peregrine* Insect Mol Biol 2004 13 133 140 15056360 10.1111/j.0962-1075.2004.00468.x

[B25] Zelensky AN Gready JE The C-type lectin-like domain superfamily FEBS J 2005 272 6179 6217 16336259 10.1111/j.1742-4658.2005.05031.x

[B26] Wang JL Liu XS Zhang Q Zhao HB Wang YF Expression profiles of six novel C-type lectins in response to bacterial and 20E injection in the cotton bollworm (*Helicoverpa armigera*) Develop Comp Immuno 2012 2 221 232 10.1016/j.dci.2012.04.004 22516747

[B27] Bartholomay LC Waterhouse RM Mayhew GF Campbell CL Michel K Zou Z Ramirez JL Das S Alvarez K Arensburger P Bryant B Chapman SB Dong YM Erickson SM Karunaratne SHPP Kokoza V Kodira CD Pignatelli P Shin SW Vanlandingham DL Atkinson PW Birren B Christophides GK Clem RJ Hemingway J Higgs S Megy K Ranson H Zdobnov EM Raikhel AS Pathogenomics of Culex quinquefasciatus and Meta-Analysis of Infection Responses to Diverse Pathogens Science 2010 330 88 90 20929811 10.1126/science.1193162 PMC3104938

[B28] Geijtenbeek TB van Vliet SJ Engering AT Hart BA van Kooyk Y Self- and nonself-recognition by C-type lectins on dendritic cells Annu Rev Immunol 2004 22 33 54 15032573 10.1146/annurev.immunol.22.012703.104558

[B29] Kanost MR Jiang H Yu XQ Innate immune responses of a Lepidopteran insect Manduca sexta Immunol Rev 2004 198 97 105 10.1111/j.0105-2896.2004.0121.x 15199957

[B30] Seufi AM Galal FH Hafez EE Characterization of Multisugar-Binding C-Type Lectin (SpliLec) from a Bacterial-Challenged Cotton Leafworm, *Spodoptera littoralis* PLoS ONE 2012 7 e42795 doi:10.1371/journal.pone.0042795 22916161 10.1371/journal.pone.0042795 PMC3423437

[B31] Rossignol PA Lueders AM Bacteriolytic factor in the salivary glands of *Aedes aegypti* Comp Biochem Physiol B 1986 83 819 822 3519067 10.1016/0305-0491(86)90153-7

[B32] Marinotti O James AA Ribeiro JMC Diet and salivation in female *Aedes aegypti* mosquitoes J Insect Physiol 1990 36 545 548

